# N-95 masks: how much do we know?

**DOI:** 10.1186/s43162-020-00014-z

**Published:** 2020-08-31

**Authors:** Satvinder Singh Bakshi, Sumita Bakshi

**Affiliations:** 1 Department of ENT and Head and Neck Surgery, AIIMS Mangalagiri, Guntur, Andhra Pradesh 522503 India; 2 Dr Smilez Dental clinic, Pondicherry, 605001 India

Dear editor,

During the present COVID-19 pandemic, personal protective equipment (PPE) has become the talk of most healthcare systems across the globe. One of the important components of PPE is the face mask.

An N95 mask also called as N95 respirator is a particulate-filtering mask that meets the US National Institute for Occupational Safety and Health (NIOSH) N95 classification of air filtration, meaning that it filters at least 95% of airborne particles of 0.3 μ or more [1]. These masks do not filter gasses and can be used against infectious particles including *Mycobacterium tuberculosis*, avian influenza, and Ebola [1]. N95 masks are usually made of four layers of melt-blown nonwoven polypropylene fabric treated to sustain an electrostatic charge. Therefore, in addition to creating a mechanical barrier against aerosols, the filters retain charged particles, such as bacteria. Depending on the standardization and country of use, these masks can have different names, for example:

1. N95 (United States National Institute for Occupational Safety and Health [NIOSH]-42CFR84)
2. Filtering facepiece particles 2 (FFP2) (Europe EN 149-2001)
3. KN95 (China GB2626-2006)
4. P2 (Australia/New Zealand AS/NZA 1716:2012)
5. Korea 1st class (Korea KMOEL-2017-64)
6. DS (Japan JMHLW-Notification 214, 2018).

American and European standards are the most commonly used standardizations across the globe. The edges of the masks are designed to form a close seal around the nose and mouth; therefore, they cannot be used for children or people with facial hair because a proper fit cannot be achieved. Sustained usage of N95 is associated with a gradual increase in blood carbon dioxide content and perceived exertion, shortness of breath, headache, and lightheadedness [2]. This makes compliance difficult especially in people with chronic respiratory or cardiac conditions. Some masks have exhalation valves that can make breathing out easier and help reduce heat build-up [3].

The most important thing in using the masks is that a “fit test” should be done in the beginning to check for the fit of the mask by using masks of different sizes. Besides every time while donning the mask a “user seal check” with either positive or negative pressure should be performed [3, 4]. Although not recommended, N95 masks can be reused a limited number of times provided that they have not been used during aerosol-generating procedures and are not contaminated with patients’ bodily fluids. The masks should always be checked for integrity before being donned especially the area of the nose bridge, nose foam, and straps. The advocated methods for decontamination include hydrogen peroxide vapors, ultraviolet irradiation, and moist heat [4]. The use of adjunct measures like the use of face shields, airflow control in wards and intensive care units, early discharge of patients can help increase the life usage of these masks and help tackle the shortage currently faced by countries worldwide.

## Data Availability

Not applicable

## Abbreviations

COVID-19: Corona virus 2019
PPE: Personal protection equipment
NIOSH: National Institute for Occupational Safety and Health
